# Lithium-Decorated C_26_ Fullerene in DFT Investigation: Tuning Electronic Structures for Enhanced Hydrogen Storage

**DOI:** 10.3390/molecules30153223

**Published:** 2025-07-31

**Authors:** Jiangang Yu, Lili Liu, Quansheng Li, Zhidong Xu, Yujia Shi, Cheng Lei

**Affiliations:** State Key Laboratory of Exterme Environment Optoelectronic Dynamic Measyrement Technology and Instrument, North University of China, Taiyuan 030051, China; yujg@nuc.edu.cn (J.Y.); 2206040245@st.nuc.edu.cn (Q.L.); 2319014148@st.nuc.edu.cn (Z.X.); 13417536993@163.com (Y.S.)

**Keywords:** density functional theory, H_2_ adsorption, C_26_ fullerene, electron transfer

## Abstract

Hydrogen energy holds immense potential to address the global energy crisis and environmental challenges. However, its large-scale application is severely hindered by the lack of efficient hydrogen storage materials. This study systematically investigates the H_2_ adsorption properties of intrinsic C_26_ fullerene and Li-decorated C_26_ fullerene using density functional theory (DFT) calculations. The results reveal that Li atoms preferentially bind to the H_5-5_ site of C_26_, driven by significant electron transfer (0.90 |e|) from Li to C_26_. This electron redistribution modulates the electronic structure of C_26_, as evidenced by projected density of states (PDOS) analysis, where the p orbitals of C atoms near the Fermi level undergo hybridization with Li orbitals, enhancing the electrostatic environment for H_2_ adsorption. For Li-decorated C_26_, the average adsorption energy and consecutive adsorption energy decrease as more H_2_ molecules are adsorbed, indicating a gradual weakening of adsorption strength and signifying a saturation limit of three H_2_ molecules. Charge density difference and PDOS analyses further demonstrate that H_2_ adsorption induces synergistic electron transfer from both Li (0.89 |e| loss) and H_2_ (0.01 |e| loss) to C_26_ (0.90 |e| gain), with orbital hybridization between H s orbitals, C p orbitals, and Li orbitals stabilizing the adsorbed system. This study aimed to provide a comprehensive understanding of the microscopic mechanism underlying Li-enhanced H_2_ adsorption on C_26_ fullerene and offer insights into the rational design of metal-decorated fullerene-based systems for efficient hydrogen storage.

## 1. Introduction

As the global energy demand soars incessantly and the alarm over environmental challenges intensifies, the quest for energy storage solutions that are both efficacious and enduring has ascended to a critical imperative [1,2]. Hydrogen, endowed with high energy density, renewability, and friendliness toward the environment, emerges as a promising clean energy carrier, making it a potential substitute for fossil fuels in the future energy landscape [3,4]. Yet, the large-scale application of hydrogen energy is limited by the difficulties in its storage and transportation, particularly the lack of efficient and reversible hydrogen storage materials at room temperature [5,6]. The efficient storage of hydrogen is a critical challenge that must be addressed to facilitate the widespread adoption of hydrogen as an energy carrier [7].

Among various hydrogen storage methods, physical adsorption on solid materials has emerged as a particularly attractive option, offering the potential for high storage capacity, rapid adsorption kinetics, and low energy consumption [8,9]. In recent years, carbon-based nanomaterials, such as carbon nanotubes, graphene, and fullerenes, have garnered significant interest for hydrogen storage because of their unique structures and properties [10,11]. Fullerenes with their hollow and symmetrical cage-like structures present a sufficient space for the physisorption of hydrogen molecules [12,13]. Compared to common fullerenes such as C_60_, the larger curvature of C_26_ may result in a more specific electron cloud distribution, thereby influencing its interaction patterns with other substances [14,15]. Meanwhile, the smaller molecular size could endow it with unique advantages in certain application scenarios, such as the fabrication of nanoscale devices or targeted delivery in the biomedical field [16,17]. Owing to its unique structural and electronic properties, C_26_ fullerene is expected to serve as a potential high-efficiency hydrogen storage material [18].

The storage capacity of pristine fullerenes, however, is limited by their weak van der Waals interactions with hydrogen molecules [19]. To enhance the hydrogen storage performance of fullerenes, various strategies have been explored, including the modification of fullerenes with metal atoms [20,21]. Metal decoration has been identified as an effective means to strengthen the interaction between fullerenes and hydrogen molecules, thereby increasing the storage capacity and reversibility of the system [22,23].

Lithium (Li), an alkali metal with a high affinity for hydrogen, stands out as a potential modifier for modifying fullerenes to enhance hydrogen storage [24]. The interaction between Li and hydrogen is strong enough to provide a significant binding energy, while the low weight of Li contributes to a high gravimetric storage capacity [25]. Moreover, the electronic structure of Li-decorated fullerenes can be tuned to optimize the hydrogen adsorption and desorption processes under mild conditions, which is essential for practical applications [26].

Therefore, it is necessary to investigate the H_2_ adsorption properties of C_26_ fullerene to understand its hydrogen storage mechanism. In this study, the adsorption sites, interaction energies, and electronic structures of H_2_ adsorbed on intrinsic C_26_ fullerene and Li-modified C_26_ fullerene were studied using density functional theory (DFT) methods to evaluate their potential as hydrogen storage media. Our work aims to contribute to the development of efficient hydrogen storage materials, which are crucial for the practical implementation of hydrogen fuel cell technologies and the transition to a sustainable energy economy.

## 2. Results and Discussion

### 2.1. Hydrogen Adsorption on C_26_ Fullerene

**Figure 1:**
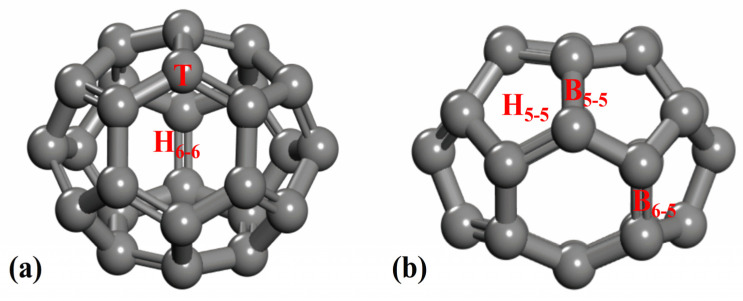
The (**a**) top and (**b**) side views of the C_26_ fullerene structure. The C atoms are shown in grey.

From the top view and side view of the C_26_ fullerene in Figure 1, we can gain insights into its structural characteristics, such as atomic arrangement and symmetry. In terms of the atomic arrangement, the C_26_ molecule consists of 26 carbon atoms, which are interconnected via covalent bonds to form a closed cage-like structure with D_3h_ symmetry. The surface of the C_26_ molecule contains three hexagonal rings and twelve pentagonal rings.

To further identify the most stable adsorption site of H_2_ on C_26_ fullerene, the different sites were considered to perform energy calculations. It was found that H_2_ exhibits the lowest adsorption energy (−0.25 eV) at the H_5-5_ adsorption site, indicating that this is the most stable adsorption site for H_2_. Upon further adsorption of hydrogen, the calculated stepwise adsorption energy is −0.01 eV, which is close to zero. This indicates that C_26_ fullerene can only adsorb one hydrogen molecule, implying that the intrinsic C_26_ fullerene is not suitable as a hydrogen carrier.

### 2.2. Li-Decorated C_26_ Fullerene

The binding characteristics of Li atoms on different sites of C_26_ fullerene were investigated, as presented in Table 1. The results reveal that the H_5-5_ site exhibits the highest binding energy (−2.68 eV) among all considered positions, indicating the most thermodynamically favorable adsorption configuration for Li decoration. This strong binding is accompanied by a relatively short C-Li distance of 2.22 Å. In contrast, the Li atom bonded at the T site is dynamically unstable, prompting Li atoms to migrate spontaneously to the H_5-5_ site, highlighting the intrinsic preference for this specific adsorption geometry. The H_6-6_ and B_5-5_ sites also demonstrate substantial binding energies (−2.59 eV and −2.42 eV, respectively), with corresponding distances of 2.28 Å and 2.11 Å, reflecting moderate interaction strengths. The B_6-5_ site, while still stable, exhibits the lowest binding energy (−2.38 eV) among the viable configurations, which is attributed to its intermediate coordination environment.

The cohesive energy of the solid bulk phase of the Li atom is 1.13 eV/atom [27], which is less than the binding energy of the Li atom on C_26_ fullerene, suggesting that the Li atom tends to bind to C_26_ fullerene rather than aggregating with each other. These findings suggest that Li-decorated C_26_ is most effective at the H_5-5_ site, which may significantly modulate the electronic structure of the C_26_ fullerene, thereby enhancing its potential as a hydrogen storage medium.

### 2.3. Interaction Between Li and C_26_ Fullerene

**Figure 2:**
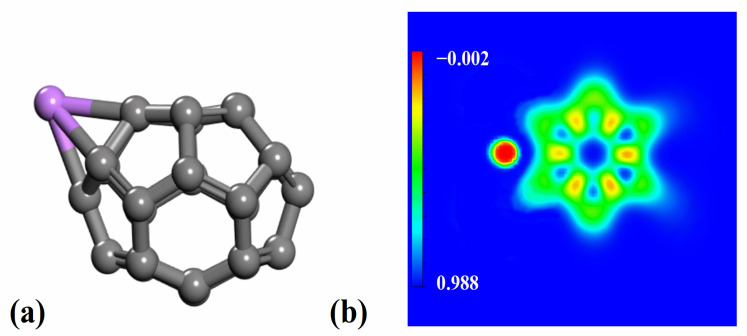
(**a**) Optimized structure and (**b**) charge density difference of Li−decorated C_26_ fullerene. The C atoms and Li atom are shown in grey and purple, respectively. Red regions indicate charge depletion, while green and blue represent regions of reduced and increased charge density, respectively. The isosurfaces are set to 0.16 eÅ^−3^.

To further explore the intrinsic relationship between the electronic structure of Li and C_26_ fullerene, the charge density difference for the Li-decorated C_26_ fullerene was studied. As shown in Figure 2b, a region of electron depletion (red) is observed around the Li atom, with a region of electron accumulation (blue) around the C_26_ fullerene. This indicates that electron transfer occurs from the Co surface to the C atom. Bader charge analysis [28,29] showed that 0.90 electrons (0.90 |e|) are transferred from Li atoms to C_26_ fullerene after the interaction between them. This charge transfer process is attributed to the electronegativity difference between Li atoms and C_26_ fullerene. Li atoms exhibited low electronegativity and weak electron-binding ability, whereas C_26_ fullerene had relatively higher electronegativity and stronger electron-attracting capacity. The increased electron cloud density of C_26_ fullerene after gaining electrons may enhance its ability to adsorb hydrogen.

**Figure 3:**
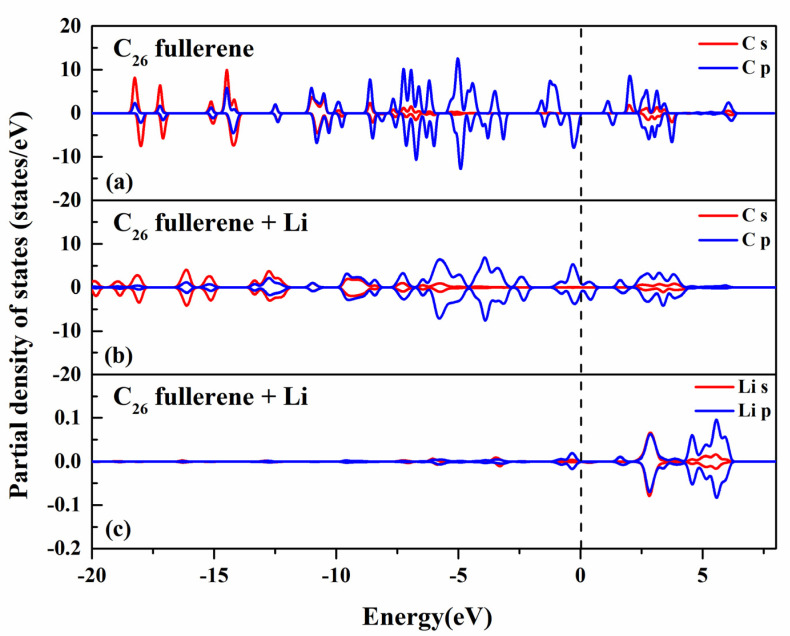
The projected density of states (PDOS) onto (**a**) s and p orbitals of the C atom of C_26_ fullerene; (**b**) s and p orbitals of the C atom of Li−decorated C_26_ fullerene; (**c**) s and p orbitals of the Li atom of Li−decorated C_26_ fullerene;. The Fermi energy is set to zero. The s and p orbitals are labeled with red and blue lines, respectively.

To gain an in-depth understanding of the interaction between Li and C_26_ fullerene, a detailed analysis was performed on the projected density of states (PDOS) of C_26_ fullerene with Li decoration. As observed in Figure 3a, the density of states C_26_ molecule is mainly composed of the s and p orbitals of the C atoms. Among these, a distinct distribution of the density of states of the p orbitals near the Fermi level was observed. This is due to the formation of a conjugated system via covalent bonding between carbon atoms in the C_26_ molecule, which allows p-orbital electrons to be delocalized within the molecule, thereby influencing the electronic properties of C_26_ fullerene.

After Li decoration, as shown in Figure 3b, the s orbital of C atoms becomes broader and more dispersed compared to pristine C_26_ fullerene. The C s orbital exhibits broadened and less intense peaks compared to pristine C_26_ fullerene, indicating electron redistribution induced by Li decoration. This effect is attributed to charge transfer from Li to C_26_ fullerene, altering the electrostatic environment around C atoms. Near the Fermi level, the C p orbital shows reduced peak intensities and increased peak widths, suggesting weakened electron delocalization. This reduction is caused by electron injection from Li, which modifies the hybridization state of C atoms. From Figure 3c, it can be seen that the s and p orbitals of the Li atom show sparse low-intensity peaks concentrated at higher energies (0 eV to 5 eV), consistent with the alkali-metal character of Li.

The PDOS analysis demonstrates that Li decoration modifies the electronic structure of C_26_ through electron transfer and weak orbital hybridization. These changes enhance the electron density on C_26_, particularly around the Fermi level, which may improve its ability to adsorb hydrogen by providing favorable electrostatic interactions and increased orbital overlap with H_2_ molecules.

### 2.4. Hydrogen Adsorption on Li-Decorated C_26_ Fullerene

**Figure 4:**
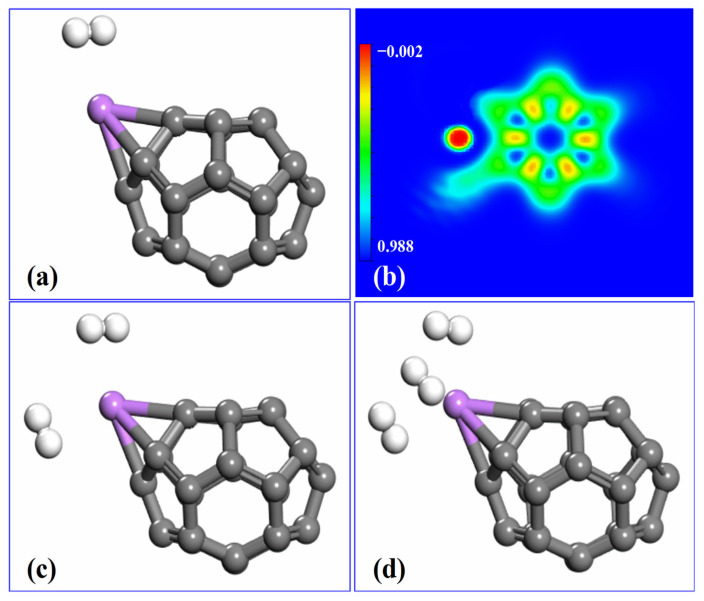
The optimized structure of Li−decorated C_26_ fullerene with (**a**) 1 H_2_ adsorption, (**c**) 2 H_2_ adsorption, and (**d**) 3 H_2_ adsorption. (**b**) Charge density difference of Li−decorated C_26_ fullerene with H_2_ adsorption. The H, C, and Li atoms are shown in white, grey, and purple, respectively. Red regions indicate charge depletion, while green and blue represent regions of reduced and increased charge density, respectively. The isosurfaces are set to 0.16 eÅ^−3^.

To investigate the role of Li in enhancing the hydrogen adsorption performance of C_26_ fullerene, the optimized structures of Li-decorated C_26_ fullerene with different numbers of adsorbed nH_2_ molecules were investigated, as depicted in Figure 4. For the structure with one H_2_ adsorption (Figure 4a), the single H_2_ molecule is positioned near the Li-C_26_ with the average adsorption energy of −0.15 eV. When two H_2_ molecules are adsorbed (Figure 4c), the second H_2_ molecule attaches to the system in a distinct orientation relative to the first one, suggesting that the surface of the Li-decorated C_26_ has additional available sites to accommodate more H_2_. As for the structure with three H_2_ adsorptions (Figure 4d), the third H_2_ molecule and the first two H_2_ molecules present a triangular symmetric position.

The average adsorption energy, consecutive adsorption energy, and Li-H_2_ distance for increasing numbers of H_2_ molecules adsorbed onto Li-decorated C_26_ fullerene are shown in Table 2. As the number of adsorbed H_2_ molecules (n) increases, the average adsorption energy shows a gradual decreasing trend, dropping from −0.15 eV when n = 1 to −0.09 eV when n = 4. This indicates that the adsorption capacity of C_26_ for H_2_ is gradually weakened.

The consecutive adsorption energy reflects the energy change during the adsorption process when one more hydrogen molecule is added. It can be observed from Table 2 that the consecutive adsorption energy gradually decreases, falling from −0.15 eV when n = 1 to −0.02 eV when n = 4. This means that as the number of already adsorbed hydrogen molecules increases, it becomes increasingly difficult to adsorb one more hydrogen molecule. When n reaches 4, the value of consecutive adsorption energy is −0.02 eV, close to zero, indicating that the maximum saturation number of hydrogen molecules adsorbed by the C_26_ structure is 3, which is more than one H_2_ adsorption of intrinsic C_26_ fullerene. The distance increases from 2.09 Å (1H_2_) to 2.44 Å (4H_2_), implying a weakening interaction between Li and H_2_ as more H_2_ molecules are adsorbed.

### 2.5. Interaction of Hydrogen with Li-Decorated C_26_ Fullerene

To elucidate the electronic effects of H_2_ adsorption, the charge density difference of H_2_ adsorption on Li-decorated C_26_ fullerene was studied, as shown in Figure 4b. The electron transfer was quantified by Bader charge analysis: after hydrogen absorption, Li loses 0.89 |e|, H_2_ loses 0.01 |e|, and C_26_ gains 0.90 |e|. Upon H_2_ adsorption, the charge depletion around Li becomes more pronounced in the H_2_ adsorbed system (broader red–green regions), indicating a strengthened electron-donating role of Li under H_2_ interaction. Conversely, the charge accumulation on C_26_ expands and intensifies (more extensive blue regions) compared to the Li-decorated C_26_ system, which is attributed to the combined electron transfer from both Li and H_2_. The decoration of Li to C_26_ leads to the spatial charge redistribution, which enhances H_2_ adsorption stability to its saturation at three H_2_ molecules, consistent with the trends in adsorption energy, providing a microscopic storage mechanism for C_26_ fullerene.

**Figure 5:**
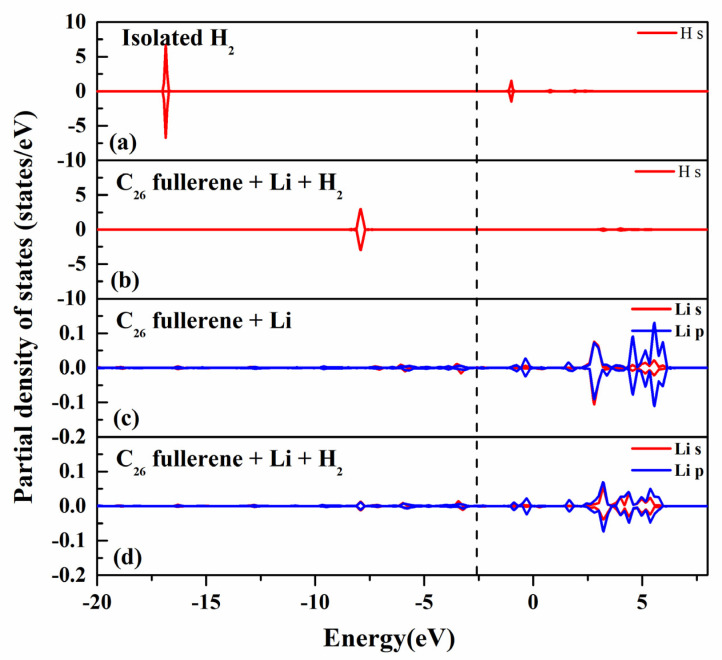
The projected density of states (PDOS) onto (**a**) s orbital of the H atom of isolated H_2_ molecule; (**b**) s orbital of the H atom of Li−decorated C_26_ fullerene after H_2_ adsorption; (**c**) s and p orbitals of the Li atom of Li−decorated C_26_ fullerene; (**d**) s and p orbitals of the Li atom of Li−decorated C_26_ fullerene after H_2_ adsorption. The Fermi energy is set to zero. The s and p orbitals are labeled with red and blue lines, respectively.

The PDOS of Li-decorated C_26_ fullerene after H_2_ adsorption was calculated to explore the interaction of H_2_ with Li-decorated C_26_ fullerene, as shown in Figure 5. For the isolated H_2_ molecule in Figure 5a, a sharp and intense peak is observed in the H s orbital at −17 eV. After H_2_ adsorption onto Li-decorated C_26_, as shown in Figure 5b, the peak of the H s orbital was shifted to a higher energy (−5 eV) and broadened. This change is attributed to electron transfer and orbital hybridization between H_2_ and the C_26_-Li system. The interaction disrupts the localized H_2_ s orbital, redistributing electron density and altering the energy distribution of H electrons.

As shown in Figure 5d, upon H_2_ adsorption, the peak intensities in s and p orbitals of the Li atom were decreased, and distributions became more dispersed. This reduction in electron density is caused by electron transfer from Li to H_2_, modifying the local electronic environment of the Li atom. The synergistic effects of electron transfer and orbital hybridization resulted in preferential H_2_ adsorption at Li-decorated C_26_, exhibiting selectivity. This selective adsorption is of great significance for the development of efficient hydrogen storage materials, as it can improve both the storage capacity and selectivity of C_26_ fullerene.

### 2.6. Kubas-Type Interaction

In our calculations, the H-H bond length of H_2_ molecules adsorbed on Li-decorated C_26_ (for one to four H_2_ molecules) was consistently 0.77 Å, which is slightly elongated compared to the 0.75 Å bond length of isolated H_2_. This slight elongation (0.02 Å) aligns with the key characteristic of Kubas-type interactions, where H-H bond length changes moderately (neither drastic enough for chemisorption nor negligible for pure physisorption), as observed in the Y-decorated C_24_ system [30].

Additionally, our charge density difference and PDOS analyses reveal electron transfer from Li and H_2_ to C_26_, with orbital hybridization between H s orbitals, C p orbitals, and Li orbitals. This orbital interaction, coupled with the observed H-H bond elongation, suggests a mechanism analogous to the Kubas interaction described for Y-decorated systems, where charge transfer and weak orbital hybridization contribute to an interaction strength between physisorption and chemisorption.

Notably, the H-H bond length remains nearly constant as more H_2_ molecules are adsorbed, while the distance between Li and H centers increases slightly. This stability in bond elongation further supports the presence of Kubas-type interactions, as the interaction mechanism remains consistent across different H_2_ coverage levels. These observations indicate that Kubas-type interactions play a significant role in H_2_ adsorption on Li-decorated C_26_, contributing to the enhanced adsorption capacity compared to intrinsic C_26_ fullerene. In future research, we will further elaborate on the orbital-level charge transfer details to better quantify this interaction.

## 3. Computational Details

### Computational Methods

Density functional theory (DFT) computations were employed by the Vienna ab initio simulation package (VASP) [31,32] 6.1.0. The interactions between electrons and ions were represented by the projector augmented wave (PAW) method [33,34]. The exchange-correlation functional was determined within the framework of the generalized gradient approximation (GGA) with Perdew–Burke–Ernzerhof (PBE) functional [35,36]. Spin polarization was calculated during the process of geometric optimization, and van der Waals (vdW) interactions were considered by the DFT-D3 method with Becke–Johnson damping [27]. A plane wave basis set cutoff energy [37] of 450 eV was applied. The convergence criteria of geometric optimization process for energy and force thresholds were set to 10^−5^ eV and 0.02 eV/Å, respectively. To separate the interactions between adjacent slabs, a 30 Å vacuum was established. The k-point integration was sampled using the Monkhorst–Pack scheme [38] with a (1 × 1 × 1) grid. The vacuum layer was set to 20 Å to avoid the interaction of periodically repeated slabs.

The binding energy (*E*_b_) of the Li atom to C_26_ was calculated as follows:

(1) $$E_{b}=E_{{\mathrm{Li}+C}_{26}}-E_{C_{26}}-E_{\mathrm{Li}}$$

where *E*_Li+C26_, *E*_C26_, and *E*_Li_ are the total energies of Li-decorated C_26_ fullerene, clean C_26_ fullerene, and isolated Li atoms, respectively. Hence, a negative binding energy suggests that the metal atom can be attached to the C_26_.

The average adsorption energy (*E*_ad_) and consecutive adsorption energy (*E*_c_) of the C_26_ fullerene to the hydrogen molecule were calculated as follows:

(2) $$E_{\mathrm{ad}}=\left(E_{{\mathrm{nH}}_{2}+{\mathrm{Li}+C}_{26}}-E_{{\mathrm{Li}+C}_{26}}-nE_{H_{2}}\right)/n$$

(3) $$E_{c}=E_{{\mathrm{nH}}_{2}+{\mathrm{Li}+C}_{26}}-E_{\left(n-1\right)H_{2}+{\mathrm{Li}+C}_{26}}-E_{H_{2}}$$

where *E_n_*_H2+Li+C26_, *E*_(n−1)H2+Li+C26_, and *E*_H2_ are the total energies of nH_2_ and (n − 1)H_2_ adsorbed on the Li-decorated C_26_ fullerene and the total energy of an isolated hydrogen molecule, respectively; n is the number of adsorbed hydrogen molecules.

The larger absolute value of adsorption energy indicates a stronger adsorption strength. The larger the negative value, the stronger the adsorption capacity. The consecutive adsorption energy is the adsorption energy when the nth hydrogen molecule adsorbed onto C_26_, which already has (n − 1) hydrogen molecules adsorbed. The small value of consecutive adsorption energy implies that the adsorption process is not energetically favored, which is typically not desired for efficient hydrogen storage materials.

## 4. Conclusions

In summary, this study systematically investigates the structural, electronic, and H_2_ adsorption properties of Li-modified C_26_ fullerenes through DFT calculations, unraveling the critical role of Li decoration in modulating H_2_ adsorption behavior at the atomic and electronic levels. Intrinsic C_26_ fullerene exhibits limited hydrogen storage capacity, with a maximum adsorption of only one H_2_ molecule due to weak van der Waals interaction. Li decoration emerges as an effective strategy to overcome this limitation. The strong binding of Li atoms to the H_5-5_ site of C_26_ triggers substantial electron transfer (0.90 |e|) from Li to C_26_. PDOS reveals that Li decoration reconstructs the s and p orbitals of C atoms, altering electron distribution near the Fermi level and strengthening interactions with H_2_. For Li-decorated C_26_, the average adsorption energy and consecutive adsorption energy both decrease with increasing H_2_ coverage, with a saturation capacity of three H_2_ molecules. This saturation is attributed to the weakening Li-H_2_ interaction and reduced electron donation from Li, as observed in the charge density. Bader charge analysis further reveals that H_2_ adsorption induces synergistic electron transfer from Li and H_2_ to C_26_, with orbital hybridization between H s orbitals, C p orbitals, and Li orbitals stabilizing the adsorbed system, as evidenced by PDOS shifts in H s orbitals. These findings demonstrate that Li decoration enhances the hydrogen storage capacity of C_26_ by modulating its electronic structure through electron transfer and orbital hybridization, providing a theoretical basis for designing C_26_-based hydrogen storage materials.

## Figures and Tables

**Table 1:** The binding energy (*E*_b_) of the Li atom on C_26_ fullerene and the distance (*d*_**C-Li**_, Å) between Li and C_26_ at different sites of Li-decorated C_26_ fullerene.

Site	*E*_b_ (eV)	*d*_C-Li_ (Å)
H_5-5_	−2.68	2.22
H_6-6_	−2.59	2.28
B_5-5_	−2.42	2.11
B_6-5_	−2.38	2.12
T	unstable and move to H_5-5_ site	

**Table 2:** The average adsorption energy (*E*_ads_), consecutive adsorption energy (*E*_c_) of the nH_2_/Li-C_26_ system, and corresponding distance (*d*_**C-Li**_, Å) between Li and H_2_.

*n*H_2_	*E*_ads_ (eV)	*E*_c_ (eV)	*d*_Li-H2_ (Å)
1H_2_	−0.15	−0.15	2.09
2H_2_	−0.13	−0.11	2.13
3H_2_	−0.11	−0.07	2.25
4H_2_	−0.09	−0.02	2.44

## Data Availability

The original contributions presented in this study are included in the article. Further inquiries can be directed to the corresponding author(s).
